# Seropositive rheumatoid arthritis in osteogenesis imperfecta type XI (FKBP10 mutation): first case report and literature review

**DOI:** 10.1186/s13023-025-04071-x

**Published:** 2026-01-13

**Authors:** Anas Manhal, Jamal Abdallah, Mahmoud M. Qouqas, Ahmad Waleed, Layth Al-Karaja, Noor Alhuda Sawalha, Laith Alamlih

**Affiliations:** 1 Faculty of Medicine, Hebron University, Hebron, 150 Palestine; 2 Al-Quds University, Abu Dis, Palestine; 3 Division of Pediatric Endocrinology, Department of Pediatrics, Al-Ahli Hospital, Hebron, West Bank Palestine; 4 Department of Pediatrics, Hebron University, Hebron, West Bank Palestine; 5 Almeezan Hospital, Halhul, Palestine

**Keywords:** Osteogenesis imperfecta, Rheumatoid arthritis, FKBP10, Rare disease, Case report, Bone fragility, Autoimmune disease

## Abstract

**Background:**

Osteogenesis imperfecta (OI) is a rare genetic disorder primarily caused by mutations in genes involved in type I collagen production. We report a 27-year-old female with genetically confirmed OI type XI (OI-XI) who experienced a delayed diagnosis of seropositive rheumatoid arthritis (RA), resulting in irreversible deformities.

**Case presentation:**

The patient had multiple congenital contractures and became wheelchair-dependent in early childhood. She received only one course of bone protection therapy in her lifetime. Two years prior to presentation, she developed bilateral hand pain, stiffness, and progressive deformities. The diagnosis of RA was confirmed based on clinical features, imaging, and high titers of anti-cyclic citrullinated peptide (anti-CCP) antibodies. Genetic analysis revealed a homozygous FKBP10 mutation (c.391 + 4 A > T), confirming OI-XI. Treatment with methotrexate, folic acid, and vitamin D led to symptom improvement and stabilization of deformities.

**Conclusions:**

This is the first reported case of RA in a patient with genetically confirmed OI-XI. The case underscores the importance of early detection and treatment of RA in individuals with OI to prevent irreversible joint damage.

**Clinical trial number:**

Not applicable.

## Introduction

Osteogenesis imperfecta (OI) is a rare skeletal dysplasia that occurs in approximately 1 in every 15,000 to 20,000 individuals [1]. OI is a heterogeneous disorder primarily caused by mutations in genes involved in the production or processing of type I collagen and is characterized by bone fragility resulting in fractures, bone deformities, and growth retardation [2]. OI can also lead to extra-skeletal manifestations such as blue sclera, dentinogenesis imperfecta (DI), joint hypermobility, hearing loss, cardiovascular and respiratory complications [3].

Traditionally, OI has been classified into four major types based on clinical phenotype according to the Sillence classification [4]. More recently, a genetic classification system based on underlying causative genes has been proposed, identifying over 20 subtypes that reflect both genetic and phenotypic heterogeneity [5, 6]. The majority of OI cases are caused by autosomal dominant mutations in COL1A1 or COL1A2, while rarer autosomal recessive forms, such as OI type XI, result from mutations in genes involved in collagen modification and folding (e.g., FKBP10) [7].

Osteogenesis imperfecta type XI (OI-XI), is caused by a bi-allelic mutation of FKBP10 gene that results in a defect of FKBP65 protein production that leads to a delayed type I procollagen secretion and accumulation in the endoplasmic reticulum [8, 9]. A distinctive clinical feature of OI XI is the presence of congenital joint contractures, which contrasts with classic forms of OI. Additionally, patients often develop progressive kyphoscoliosis. Interestingly, features commonly seen in other OI types, such as blue sclera, dentinogenesis imperfecta, and hearing loss are uncommon in this subtype [8, 10].

Rheumatoid arthritis (RA) is a common chronic inflammatory disorder marked by inflammation of the synovial membrane within the joints. This persistent inflammation ultimately if untreated leads to the destruction of joint components, including cartilage and bone, resulting in significant damage to the articular structures over time [11]. RA affects approximately 1% of the global population, with an annual incidence rate of about 3 per 10,000 adults. The condition is two to three times more prevalent in women than men. About one-third of RA patients develop disabilities, depending on several factors including the disease’s initial severity, time of diagnosis and commitment to treatment and follow up. Although the exact cause of RA remains unclear, genetic factors contribute up to 30% of disease susceptibility. Key genetic links include HLA-DR4 and DR1, which share specific regions that increase the risk of developing RA. Various infectious agents have also been proposed as potential triggers [12].

The coexistence of osteogenesis imperfecta and inflammatory arthritis is rare, but systemic inflammation has been found in osteogenesis imperfecta. Mutations in COL1A1 can affect collagen synthesis and interactions with the extracellular matrix, including ligands involved in rheumatoid arthritis [13]. Notably, a study by McKiernan et al. examining 111 patients with type 1 OI found that while nearly half had noninflammatory arthritis, such as osteoarthritis, only a small proportion (4%) were diagnosed with inflammatory arthritis, including psoriatic and rheumatoid arthritis [14].

In this case report, we describe what we believe to be the first documented instance of RA in a patient with genetically confirmed OI-XI—highlighting the diagnostic and clinical challenges involved.

## Methods (case description)

### Patient information

The patient is a 27-year-old woman born with multiple congenital contractures involving her knees, ankles, and—less severely—her elbows and hands. She achieved independent ambulation with a tiptoe gait by age one and underwent several tendon lengthening procedures early in life. In 2000, at the age of four, she sustained her first low-trauma femoral fracture, which was managed with bilateral intramedullary rodding. In 2003, at age seven, she sustained a tibial fracture that was treated with external fixation. In 2008, at age 12 years old, she received a single infusion of bisphosphonate, pamidronate, but later suffered another femoral fracture, requiring further surgery.

Following her last major surgery in 2008, she remained fracture-free but reported intermittent hip pain. Due to poor follow-up and limited resources, our patient remained off bone-protective therapy; having received it only once in her lifetime, and she did not have access to appropriate physiotherapy or essential nursing management. Although she developed progressive kyphoscoliosis, she retained normal hearing, dentition, and speech. She maintained independence in her daily activities, even though she required a wheelchair. A strong family history of OI, combined with genetic testing, confirmed a homozygous FKBP10 mutation (c.391 + 4 A > T), leading to a definitive diagnosis of OI-XI.

### Diagnostic assessment

The patient reported normal hand and arm function until the age 25 years. Between 2022 and early 2024, she reported bilateral hand pain, prolonged morning stiffness lasting over an hour, and progressive deformities. Initially attributing these symptoms to her underlying OI, she delayed seeking medical advice. However, her hand function continued to decline, eventually affecting her independence.

**Fig. 1:**
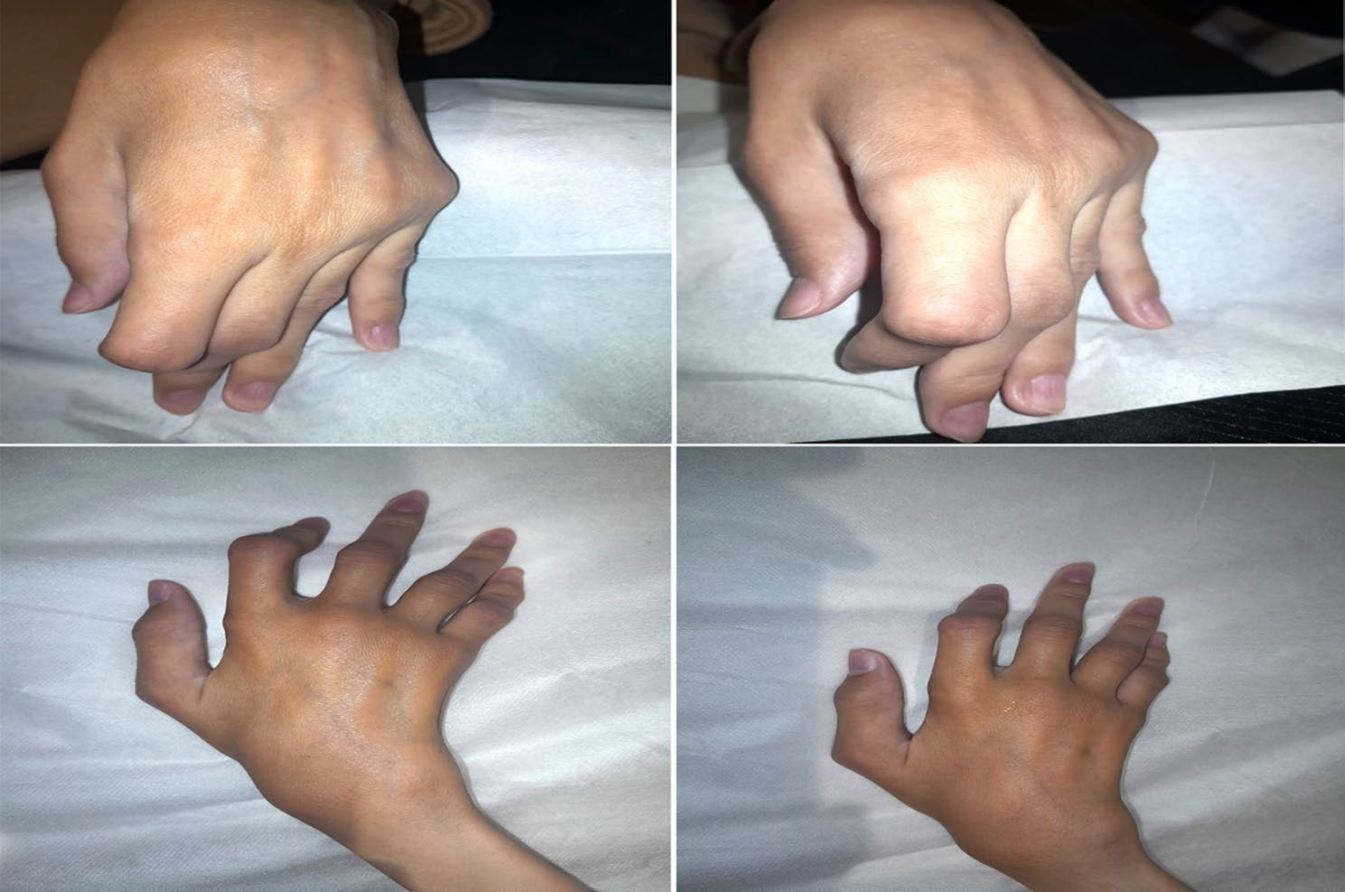
Photograph of the patient’s hands with deformities

**Fig. 2:**
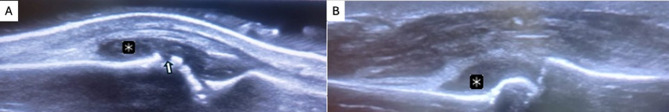
Musculoskeletal ultrasound shows (**A**) Longitudinal scan of the second Metacarpophalangeal joint (MCP) shows synovial effusion (asterisks) and bony erosion (arrow). (**B**) Longitudinal scan of the third Proximal interphalangeal joints (PIP) shows synovial effusion (asterisks)

On examination in April 2024, she measured 1.20 m in height and weighed 23 kg. She had mild blue sclerae and severe kyphoscoliosis. Examination of her hands revealed classical signs of inflammatory arthritis, including ulnar deviation and swan-neck deformities (Fig. 1), tenderness of the MCP and PIP joints, and a limited range of motion. Musculoskeletal ultrasound demonstrated joint effusions and erosions, particularly in the second MCP joints bilaterally (Fig. 2).

A DXA scan revealed severe osteoporosis with a lumbar spine BMD T-score of − 8.5 SD. Due to surgical implants, femoral measurements were not interpretable. The patient’s Disease Activity Score 28 for RA with ESR (DAS28-ESR) was 5, indicating moderate disease activity. Laboratory studies showed high titers of anti-CCP antibodies (596.7 U/mL), a positive rheumatoid factor (RF), an elevated CRP of 48 mg/L, and an ESR of 33 mm/h. All other laboratory values—including CBC, renal and liver function tests, and bone metabolism markers—were within normal limits, except for a low vitamin D level. Laboratory findings are summarized in Table 1.

**Table 1:** Laboratory results at presentation

Test Item	Patient Value (Unit)	Reference Range
**Electrolytes & Minerals**		
Total calcium	9.6 mg/dL	8.5–10.2 mg/dL
Phosphorus	2.93 mg/dL	1.5–6.8 mg/dL
Magnesium	1.7 mg/dL	1.7–2.2 mg/dL
Sodium	138 mEq/L	135–145 mEq/L
Potassium	4.6 mEq/L	3.5–5.0 mEq/L
Chloride	106 mEq/L	98–107 mEq/L
Albumin	3.97 g/dL	3.5–5.0 g/dL
Uric acid	2.00 mg/dL	2.4–6.0 mg/dL
**Renal & Liver Function**		
ALT (alanine aminotransferase)	26 U/L	7–35 U/L
AST (aspartate aminotransferase)	25 U/L	10–34 U/L
BUN (blood urea nitrogen)	14 mg/dL	7–20 mg/dL
Creatinine	0.6 mg/dL	0.5–1.1 mg/dL
**Hematology**		
WBC (white blood cells)	6.9 × 10⁹/L	4.0–10.5 × 10⁹/L
**HGB (hemoglobin)**	**11.9 g/dL**	12.0–15.5 g/dL
**HCT (hematocrit)**	**33.9%**	36.0–46.0%
**MCV (mean corpuscular volume)**	**72.7 fL**	80–100 fL
Ferritin	35 ng/mL	12–150 ng/mL
Vitamin B12	426 pg/mL	200–900 pg/mL
**Inflammatory Markers**		
**CRP (C-reactive protein)**	**48 mg/L**	< 3.0 mg/L
**ESR (erythrocyte sedimentation rate)**	**33 mm/hr**	0–20 mm/hr
**Endocrine**		
Thyroid stimulating hormone	2.42 mIU/L	0.4–4.0 mIU/L
**Vitamin D**	**15.3 ng/mL**	20–50 ng/mL
**Disease-Specific**		
**Anti-CCP (anti-cyclic citrullinated peptide)**	**596.7 U/mL**	< 20 U/mL

Abnormal values are shown in bold

Abbreviations: HGB, hemoglobin; HCT, hematocrit; MCV, mean corpuscular volume; CRP, C-reactive protein; ESR, erythrocyte sedimentation rate; ALT, alanine aminotransferase; AST, aspartate aminotransferase; BUN, blood urea nitrogen; WBC, white blood cells; Anti-CCP, anti-cyclic citrullinated peptide antibody

### Interventions

In April 2024, the patient was started on vitamin D3 (cholecalciferol) supplementation in preparation for bisphosphonates. Based on her clinical presentation, laboratory findings, and imaging, she was diagnosed with seropositive RA. Methotrexate (20 mg/week orally) in addition to folic acid were initiated in line with international guidelines for moderate RA. A short course of low-dose prednisone (5 mg daily) was prescribed and tapered off within one month as bridging therapy to rapidly control inflammation and ease the patient’s severe functional limitation while awaiting the therapeutic effect of methotrexate. The risks and benefits of glucocorticoid use were discussed in detail with the patient, given her severe osteoporosis secondary to OI-XI.

### Outcomes

In the period from 2024 to 2025, the patient responded well to treatment. Over a 10-month follow-up period, her symptoms improved significantly, and further joint deformities were halted. Repeat musculoskeletal ultrasound showed resolution of joint effusions. Her ESR decreased to 7 mm/h, and her DAS28-ESR improved from 5 to 2.3, indicating disease remission.

## Results

The clinical course, diagnostic evaluations, and treatment outcomes are outlined above. For comparison, Table 2 summarizes previously published cases of RA in patients with other types of OI.

**Table 2:** Clinical and genetic features of reported cases of osteogenesis imperfecta with coexisting inflammatory arthritis

Article / Year	Age	Sex	Serology (RF/ACPA/ESR/CRP)	Gene Mutation	Delay to Diagnosis	Major Deformities	Treatment(s)	Treatment Outcome
Our case	27	F	RF+, ACPA 596.7 U/mL, ESR 33, CRP 48	FKBP10 c.391 + 4 A > T (homozygous)	2 years	Bilateral hand deformities (ulnar deviation, swan-neck), severe kyphoscoliosis	Methotrexate, glucocorticoids, NSAIDs, vitamin D	Pain improved, deformity progression halted, inflammatory markers improved (10 months)
Bica et al., 2013 [19]	53	F	RF+, ESR 72	Clinical OI (no genetic test)	13 years (JIA at 15)	Severe hand/foot deformities, TMJ limitation	Alendronate, salicylates (irregular), steroids	Bone mass stabilized, partial pain relief, persistent TMJ stiffness, incomplete arthritis control
Damian et al., 2020 [17]	46	F	RF 28 IU/dL, ACPA 224, ESR 43, CRP 24	COL1A1 c.3399del, p.Ala1134Profs*105	6 months	Periarticular osteoporosis, tibiotarsal synovitis	Sulfasalazine, methotrexate, leflunomide	Pain and stiffness resolved, inflammation controlled, no NSAIDs/steroids used
Damian et al., 2020 [17]	70	F	RF 32, ACPA 218, ESR 34, CRP 12	COL1A1 c.3399del, p.Ala1134Profs*105	2 years	Bone deformities, periarticular bone loss	Methotrexate, leflunomide	Pain and stiffness resolved, inflammation controlled, no NSAIDs/steroids used
Mormile et al., 2022 [20]	43	F	Seropositive RA (labs N/R)	COL1A1 c.3399del, p.(Ala1134Profs*105)	N/R	N/R	Adalimumab (anti-TNF)	Complete remission

Abbreviations: RF: rheumatoid factor; ACPA: anti-cyclic citrullinated peptide antibody; ESR: erythrocyte sedimentation rate; CRP: C-reactive protein; TMJ: temporomandibular joint; JIA: juvenile idiopathic arthritis; N/R: not reported

## Discussion

Osteogenesis imperfecta (OI) patients commonly experience joint pain, stiffness, and instability, particularly in the weight-bearing joints of the lower extremities, the occurrence of symptomatic inflammatory joint disease alongside OI is rare. A study by McKiernan et al. revealed that almost half of 111 type 1 OI patients reported a diagnosis of “noninflammatory arthritis,” typically osteoarthritis. In contrast, only 4% of patients had inflammatory arthritis, such as psoriatic or rheumatoid arthritis. To date, only four cases of rheumatoid arthritis associated with OI have been reported [14]. In these patients, the time between the onset of RA symptoms and diagnosis was delayed ranging from 6 months to 13 years. Our patient suffered for two years from inflammatory joint pain until she was seen at our clinic where her evaluation revealed the seropositive rheumatoid arthritis diagnosis.

The presence of her OI-XI disease has resulted in significant delay in seeking medical advice which unfortunately resulted in severe deformities and significant loss of hands function. It is uncertain whether the presence of OI has accelerated the progression of our patient’s deformities, but this should consider, as it only took two years of joint inflammation to cause advanced deformities, whereas such changes typically take many years to develop in cases of RA [15]. This might be partly explained by systemic inflammation observed in some OI patients. In patients with OI-XI who carry an FKBP10 gene mutation, which encodes the immunophilin FKBP65, a chaperone that participates in type I procollagen folding, this mutation affects type I collagen folding and cross-linking [8]. Collagen-binding ligands, including cytokines, cell adhesion molecules, matrix metalloproteinases, proteoglycans, and other molecules, are autoimmunity targets in rheumatoid arthritis pathogenesis [13, 16]. While this could explain the coexistence of RA and OI, it does not account for the rarity of such an occurrence.

The delayed diagnosis of rheumatoid arthritis (RA) in patients with osteogenesis imperfecta (OI) can have significant adverse sequalae, including permanent deformities. RA can lead to irreversible joint damage and deformities if not promptly recognized and managed [17]. The Management of RA in patients with OI requires a comprehensive approach, involving rheumatologists, endocrinologists, orthopedic surgeons, physiotherapists and other healthcare professionals.

Treatment typically includes disease-modifying antirheumatic drugs (DMARDs) to suppress the autoimmune response, optimizing bone mineral health, bone targeted therapy (e.g. bisphosphonate), physiotherapy to improve and preserve joint function, and possibly surgical intervention to correct deformities [18].

### Differential diagnosis

In a patient with OI, symptoms such as joint pain and deformity might easily be attributed to chronic arthropathy, contractures, or secondary osteoarthritis. However, in this case, features like prolonged morning stiffness, joint swelling, high anti-CCP titers, and erosive joint changes were all characteristic of inflammatory arthritis—leading to the correct diagnosis of RA.

### Limitations

This is a single case report, and while it provides a valuable teaching point, its findings cannot be generalized. Nonetheless, it raises awareness of a potential diagnostic oversight in rare disease populations.

The rapid progression of deformities in this patient raises the question of whether OI-XI may have amplified the inflammatory process. Systemic inflammation and impaired collagen processing due to FKBP10 mutations might create an environment that facilitates autoimmunity. While speculative, this idea is consistent with emerging evidence of extracellular matrix involvement in RA pathogenesis.

Early identification and treatment of RA in patients with OI are essential to avoid functional decline. These patients require a coordinated, multidisciplinary approach involving rheumatology, orthopedics, endocrinology, and physiotherapy. Methotrexate remains the first-line DMARD and has shown good tolerability in this case. Vitamin D and bone health optimization are also key.

## Conclusions

This case represents the first known instance of seropositive RA in a patient with genetically confirmed OI-XI. It illustrates the challenges of diagnosing inflammatory arthritis in the context of pre-existing skeletal dysplasia and underscores the consequences of diagnostic delay. Clinicians should maintain a high index of suspicion for RA in any patient with new joint symptoms—even when another musculoskeletal diagnosis is already present. This case emphasizes the need to reassess chronic musculoskeletal complaints in OI patients, particularly when signs of inflammatory arthritis emerge. Early diagnosis and intervention are essential to preserving quality of life and preventing disability.

## Data Availability

All data generated or analyzed during this study are included in this published article.

## Abbreviations

OI: Osteogenesis imperfecta
DI: Dentinogenesis imperfecta
OI-XI: Osteogenesis imperfecta type XI
RA: Rheumatoid arthritis
DMARDs: Disease-modifying antirheumatic drugs
BMD: Bone mineral density
DXA: Dual-energy X-ray absorptiometry
CRP: C-reactive protein
ESR: Erythrocyte sedimentation rate
RF: Rheumatoid factor
Anti-CCP: Anti-cyclic citrullinated peptide
